# Epigenetic and Neurogenomic Mechanisms Linking Physical Activity to Brain Plasticity and Cognitive Function

**DOI:** 10.3390/genes17040474

**Published:** 2026-04-17

**Authors:** Agata Leońska-Duniec

**Affiliations:** Faculty of Physical Education, Gdansk University of Physical Education and Sport, 80-336 Gdansk, Poland; agata.leonska-duniec@awf.gda.pl

**Keywords:** neurogenomics, neuroplasticity, epigenetic regulation, transcriptomics, BDNF, physical activity, brain health, personalized exercise medicine

## Abstract

**Background/Objectives**: Physical activity is one of the most powerful lifestyle factors influencing brain health, with growing evidence supporting its role in promoting neuroplasticity, cognitive function, and resilience to age-related neurological decline. Recent studies indicate that these effects are mediated by coordinated molecular responses involving epigenetics, activity-dependent gene expression, metabolic adaptation, and inter-organ communication pathways. This narrative review synthesizes current knowledge from experimental and clinical studies on the neurogenomic and epigenetic mechanisms underlying exercise-induced brain plasticity. **Methods**: Literature searches were conducted in PubMed, Scopus, Web of Science, and Google Scholar to identify studies examining neurogenomic and epigenetic mechanisms underlying neuroplasticity and cognitive adaptations in response to exercise, with an emphasis on mechanistic and translational evidence. **Results**: Available evidence, derived predominantly from animal studies and supported by more limited, often indirect human data, indicates that physical activity induces epigenetic modifications, including changes in DNA methylation, histone modifications, and microRNA expression, which contribute to lasting changes in exercise-responsive genes involved in brain plasticity. These adaptations include the upregulation of key neuroplasticity-related mediators that support neurogenesis, synaptic plasticity, angiogenesis, and metabolic adaptation, alongside the downregulation of pathways linked to neuroinflammation, oxidative stress, and apoptotic signalling. **Conclusions**: Integrating neurogenomics with systems biology approaches offers promising opportunities to better understand how physical activity influences brain plasticity throughout life. These insights may support the development of personalized exercise medicine to improve cognitive health and reduce the risk of neurodegenerative disorders.

## 1. Introduction

The human brain shows considerable plasticity, enabling structural and functional adaptations in response to environmental stimuli, including physical activity. Regular exercise has been shown to influence multiple molecular pathways in the central nervous system (CNS), leading to changes in gene expression, cellular metabolism, and neuronal signalling [1,2,3]. In both animal and human studies, exercise has been associated with structural and functional changes in brain regions involved in memory and executive function, including the hippocampus, prefrontal cortex, and striatum [4,5]. These adaptations can be viewed within a neurogenomic framework that integrates epigenetic, transcriptional, and metabolic regulation of brain function in response to physical activity.

Recent research increasingly points to the role of epigenetic mechanisms in exercise-induced neuroplasticity. Changes in DNA methylation, histone modifications, and expression of non-coding RNA (ncRNAs) may help explain how physical activity produces longer-term effects on brain plasticity and cognitive functions [3]. These regulatory processes are accompanied by coordinated changes in neuronal gene expression, with some molecular programs being activated to support neurogenesis, synaptic plasticity, angiogenesis, and metabolic adaptation, while others, particularly those linked to neuroinflammation, oxidative stress, and apoptotic signalling, are suppressed. Consistent with this, transcriptomic studies suggest that physical activity reshapes complex gene networks in the brain, highlighting the interactions between neuronal, metabolic, and vascular mechanisms [6,7,8].

Despite substantial progress in this area, the molecular mechanisms linking exercise to the long-term regulation of brain gene expression remain incompletely understood. A particular challenge is to explain how epigenetic regulation, transcriptomic responses, and exercise-induced signalling pathways interact within an integrated neurogenomic framework [3,7]. Although the literature on physical activity and brain health is growing, related reviews have often focused on selected components of this response, such as neurotrophic signalling, epigenetic regulation, or cognitive outcomes, rather than integrating these mechanisms within a broader neurogenomic framework. The present study was therefore designed to provide a conceptually integrated synthesis of how physical activity influences brain plasticity and cognitive function through interacting epigenetic, transcriptional, metabolic, and molecular pathways. Moreover, the existing literature has largely emphasized the activation of pro-plasticity pathways, whereas the suppression of maladaptive molecular programs has received comparatively less attention [3]. An additional limitation is that most mechanistic insights derive from animal models, while direct human evidence remains relatively limited and is often indirect [5].

Therefore, the aim of this narrative review is to summarize current knowledge on the epigenetic and neurogenomic mechanisms through which physical activity influences brain plasticity and cognitive function. Brain plasticity and cognitive function are closely related but not equivalent outcomes, and the available literature does not always evaluate both within the same study design or exercise context. Accordingly, the present review integrates evidence across mechanistic, structural, and functional levels, while recognizing that epigenetic, neurobiological, and cognitive findings are not always directly aligned within individual studies. Particular attention is given to molecular pathways that regulate epigenetic modifications, gene expression, and neuronal signalling, mediating exercise-induced adaptations in the CNS. In addition, this review highlights current research gaps and discusses future directions, including the integration of multi-omics approaches, single-cell transcriptomics, and systems biology strategies to better understand the molecular basis of brain adaptations. A key point emerging from the current literature is that exercise-induced brain plasticity reflects not only the activation of adaptive molecular pathways but also the coordinated suppression of maladaptive processes that may limit neuronal resilience and synaptic remodeling.

## 2. Literature Search and Methodological Approach

This narrative review is based on a targeted literature search performed in PubMed, Scopus, Web of Science, and Google Scholar databases. Publications from January 2000 to March 2026 were considered, with particular emphasis on recent studies that reflect advances in neurogenomics, transcriptomics, and epigenetic profiling technologies relevant to brain plasticity and cognitive function. In selected cases, earlier studies were intentionally included when they provided seminal descriptions of the molecular mechanisms or conceptual frameworks addressed in this review. Search strategies combined keywords and Boolean operators related to physical activity and exercise, neuroplasticity and brain function, and epigenetic regulation. Example search terms included exercise, physical activity, brain plasticity, epigenetics, DNA methylation, histone modification, microRNA (miRNA), ncRNA, neurogenesis, and neurogenomics. Additional terms associated with molecular signalling pathways involved in neuronal adaptation, such as brain-derived neurotrophic factor (BDNF), cAMP response element-binding protein (CREB), peroxisome proliferator-activated receptor gamma coactivator 1-alpha (PGC-1α), insulin-like growth factor 1 (IGF-1), vascular endothelial growth factor (VEGF), and fibronectin type III domain-containing protein 5/irisin (FNDC5/irisin), were also incorporated where relevant. The selected literature was evaluated based on scientific significance, methodological quality, and contribution to understanding the molecular mechanisms linking physical activity with brain plasticity and cognitive outcomes. As a narrative review, the present work does not follow a formal systematic review protocol, such as the Preferred Reporting Items for Systematic Reviews and Meta-Analyses (PRISMA). Instead, it aims to synthesize current knowledge from experimental, clinical, and translational studies in order to identify major research gaps and emerging directions in the neurogenomic regulation of exercise-induced brain adaptations. Although the review was not intended to provide an exhaustive systematic appraisal of all available evidence, the literature search and article selection were guided by predefined thematic and methodological criteria in order to improve transparency and reduce the risk of selective coverage. The inclusion criteria were as follows: (i) articles addressing neurogenomic, epigenetic, molecular, or translational mechanisms linking physical activity or exercise with brain plasticity, neurogenesis, or cognitive outcomes; (ii) articles written in English; and (iii) full-text, peer-reviewed publications of clear relevance to the scope of this narrative review. Articles not directly related to exercise-induced brain adaptations, lacking neurogenomic or epigenetic relevance, or providing insufficient mechanistic insight were excluded. Conference abstracts, opinion papers, letters, theses, and book chapters were not included. This approach allowed the integration of findings across experimental, clinical, and translational domains, but may also be associated with selective coverage and limited representation of contradictory evidence.

## 3. Brain Plasticity and Exercise

Brain plasticity refers to the CNS’s ability to modify its structure and function in response to internal and external stimuli. This adaptive capacity includes processes such as adult neurogenesis, synaptic remodeling, dendritic branching, and reorganization of neuronal networks. Among various environmental factors influencing neural plasticity, physical activity has emerged as one of the strongest physiological stimuli capable of inducing structural and functional adaptations in the brain [1,4]. In this section, the term brain plasticity is used in a broad sense, encompassing structural, cellular, and functional adaptations described in both animal and human studies. However, the mechanistic depth of the available evidence differs substantially across species, and direct extrapolation of molecular findings from animal models to humans should be made with caution.

Animal studies consistently demonstrate that physical activity promotes brain plasticity through a combination of structural and molecular adaptations, whereas human evidence is stronger for structural, functional, and cognitive correlates than for direct molecular mechanisms. In animal models, exercise has been shown to stimulate hippocampal neurogenesis and angiogenesis, processes supported by molecular mediators such as IGF-1 and VEGF [9,10]. Human studies further indicate that regular exercise can induce measurable structural changes in the brain. For example, a landmark randomized controlled trial demonstrated that one year of aerobic training in older adults increased hippocampal volume and improved spatial memory performance [4]. At the molecular level, many of these adaptations are associated with activity-dependent neurotrophic signalling, particularly involving BDNF and related metabolic pathways that link skeletal muscle activity to brain function [11]. The molecular and neurogenomic mechanisms underlying these adaptations are discussed in detail in the following section, ‘Neurogenomic Signalling Pathways’.

Evidence from both animal and human studies suggests that different training programs may influence neuroplastic processes through partly distinct physiological pathways. Endurance (aerobic) exercise appears to be particularly effective at stimulating molecular mechanisms associated with hippocampal plasticity, including increased BDNF protein expression and activation of metabolic signalling pathways involving PGC-1α and FNDC5. Both acute and chronic aerobic exercise have been shown to significantly increase circulating and brain BDNF levels, supporting neuronal survival, synaptic plasticity, and cognitive function [12,13]. In contrast, resistance or strength training may exert its effects through slightly different mechanisms. Although resistance exercise can also increase BDNF levels, the response is often more temporary and may depend on training intensity and recovery dynamics [14]. Moreover, resistance training has been associated with improvements in executive functions and memory performance in older adults, suggesting that neuromuscular adaptations and increased IGF signalling may also contribute to exercise-induced neuroplasticity [15,16,17]. A meta-analytic study demonstrates that long-term aerobic training tends to produce more consistent increases in resting BDNF concentrations compared with resistance training alone [18]. For example, acute high-intensity exercise may induce marked short-term increases in circulating BDNF, whereas longer-term aerobic training appears to produce more consistent resting adaptations, suggesting that the neurobiological effects of exercise are influenced by both intensity and chronicity. Acute exercise appears to trigger rapid but transient neurobiological responses, whereas repeated training over time may promote more stable adaptations at the molecular, structural, and functional levels [18,19]. These findings suggest that while both endurance and resistance exercise can promote brain plasticity, aerobic exercise may preferentially target neurotrophic and metabolic signalling pathways linked to hippocampal neurogenesis, whereas resistance training may influence cognitive function through complementary neuromuscular and systemic adaptations. Recent studies indicate that high-intensity interval training (HIIT) may represent another potent stimulus for neuroplastic adaptations. HIIT involves repeated short bouts of high-intensity exercise interspersed with recovery periods and produces substantial metabolic and neuroendocrine responses. Human and animal evidence suggests that HIIT can induce pronounced increases in circulating BDNF, sometimes exceeding those observed after moderate-intensity continuous exercise [19]. Elevated lactate concentrations generated during high-intensity exercise may play an important signalling role, as it can cross the blood–brain barrier and stimulate BDNF protein expression in the hippocampus, thereby contributing to synaptic plasticity and cognitive enhancement [20]. In addition, interval-based training has been associated with improvements in executive function and memory performance in both young and older adults, suggesting that intense intermittent exercise may effectively engage neurobiological pathways supporting brain plasticity [21,22].

These findings demonstrate that while multiple forms of exercise can promote brain plasticity, different training programs may activate partly different physiological and molecular pathways. While these structural and functional effects of exercise are increasingly well documented, the molecular mechanisms that sustain them over time are likely to depend, at least in part, on epigenetic regulation. These observations suggest that the neurobiological effects of exercise are unlikely to be explained by a single pathway, but rather reflect the integration of multiple molecular and physiological pathways. In humans, evidence for exercise-induced brain plasticity is supported mainly by neuroimaging, cognitive, and circulating biomarker data, whereas direct molecular evidence from brain tissue remains very limited. This distinction should be considered when interpreting the translational relevance of mechanistic findings discussed in later sections.

## 4. Epigenetic Mechanisms Activated by Exercise

Epigenetic regulation represents a significant mechanism through which environmental stimuli, including physical activity, can induce long-lasting changes in gene expression without altering the underlying DNA sequence. These processes include DNA methylation, post-translational histone modifications, and ncRNA-mediated regulation of gene expression. They play a crucial role in neuronal plasticity, learning, and memory by modulating transcriptional activity in response to neuronal stimulation and metabolic signals, and may partly explain why different training programs produce distinct molecular and cognitive adaptations [23,24]. It should also be noted that acute and chronic exercise may induce partly distinct epigenetic responses, although this distinction remains insufficiently characterized in the current literature, particularly in relation to brain-specific mechanisms. In addition to epigenetics, genetic polymorphisms in neurotrophin-related genes may also contribute to variability in exercise responses. For instance, the *BDNF* rs6265 polymorphism has been examined in relation to metabolic and body composition adaptations following a 12-week aerobic training program [25]. The epigenetic responses discussed below should not be interpreted as uniformly elicited by all forms of exercise, as available evidence is often modality-specific and derives from heterogeneous paradigms, including voluntary wheel running, forced treadmill exercise, endurance training, and other intervention models.

One of the best-characterized epigenetic mechanisms associated with exercise is DNA methylation, which occurs mainly at cytosine residues within cytosine–phosphate–guanine (CpG) dinucleotides and can either facilitate or repress transcription, depending on the genomic context. Notably, mature neurons also show substantial levels of non-CG methylation (mCH, where H = A, C, or T). This form of methylation accumulates during postnatal neuronal maturation and has been linked to cell-type-specific transcriptional regulation, highlighting the complexity of DNA methylation dynamics in the CNS [26]. Changes in DNA methylation patterns have been observed in response to physical activity in both peripheral tissues and the CNS, predominantly in animal studies. In general, reduced methylation in promoter regions is associated with transcriptional activation, whereas increased methylation may contribute to transcriptional repression [27,28]. These processes are regulated by DNA methyltransferases (DNMTs), which establish and maintain methylation patterns, and by ten–eleven translocation (TET) proteins, which promote active DNA demethylation by oxidizing 5-methylcytosine. In the brain, both enzyme families are involved in activity-dependent gene regulation and neuronal plasticity [29]. The methyl-CpG-binding protein (MeCP2) also acts as an important reader of DNA methylation, linking methylation marks to transcriptional regulation and playing a critical role in neuronal function and brain development [30]. Specifically, exercise has been linked to DNA demethylation at *Bdnf* promoter IV in the rat hippocampus, accompanied by increased protein expression, supporting a role for methylation dynamics in the upregulation of neuroplasticity-related genes [31]. By contrast, in peripheral inflammatory cells, chronic moderate exercise has been associated with increased methylation of the *ASC* (apoptosis-associated speck-like protein containing a CARD) gene promoter, a change interpreted as consistent with reduced protein expression and attenuation of pro-inflammatory signalling [32].

Histone modifications represent another important epigenetic mechanism regulating gene transcription during neuronal plasticity. Acetylation of histone proteins generally promotes transcriptional activation by loosening chromatin structure and increasing transcription factor accessibility to DNA. Previous rodent studies have demonstrated that histone acetylation within the hippocampus increases following physical activity, which is associated with enhanced expression of genes involved in synaptic plasticity and memory formation [3,33]. More specifically, exercise-related histone acetylation in the hippocampus has been reported at several lysine residues, including H3K9, H3K14, H4K5, H4K8, and H4K12, highlighting the site-specific nature of chromatin remodeling associated with neuronal plasticity and memory-related gene regulation [34,35]. For example, rodent studies have shown that exercise increases histone H3 acetylation at the *Bdnf* promoter in the hippocampus, accompanied by higher mRNA and BDNF protein levels, suggesting that chromatin remodeling contributes directly to the transcriptional activation of this key neuroplasticity-related gene [31,36]. In addition, Sleiman et al. demonstrated that β-hydroxybutyrate, a metabolite elevated during prolonged exercise, promotes hippocampal *Bdnf* expression by inhibiting histone deacetylase 2 and 3 (HDAC2 and HDAC3), providing a mechanistic link between exercise-related metabolism, chromatin remodeling, and neurotrophic gene activation [37]. In addition to acetylation, other histone modifications, including methylation and phosphorylation, contribute to the regulation of neuronal gene expression, synaptic plasticity, and memory-related processes in the brain. For example, histone methylation marks such as H3K4me3 and H3K27me3, as well as phosphorylation of histone H3 at serine 10 (H3S10ph), have been implicated in activity-dependent chromatin remodeling and transcriptional regulation relevant to neuronal plasticity [38].

Another regulatory layer involves ncRNAs, particularly miRNAs, which modulate gene expression at the post-transcriptional level. Physical activity has been shown to alter the expression of specific miRNAs linked to neuronal development, synaptic function, stress regulation, and neuroinflammation, with much of the brain-specific evidence currently coming from animal models [39,40]. For example, voluntary exercise in SAMP8 mice was associated with reduced hippocampal miR-132 expression, improved cognition, and reduced neurodegeneration, suggesting that exercise-related modulation of miR-132 may contribute to hippocampal plasticity in aging-related cognitive decline [41]. In another study, voluntary exercise decreased hippocampal miR-124 expression while increasing glucocorticoid receptor (*Nr3c1*) expression, supporting a role for miR-124 in stress-related regulation and hippocampal adaptation [42]. Exercise has also been reported to reduce the injury-induced elevation of miR-21 in the hippocampus after traumatic brain injury, which was associated with improved spatial learning and memory, pointing to a possible role for miR-21 in neuroinflammatory and structural remodeling processes [43].

**Figure 1:**
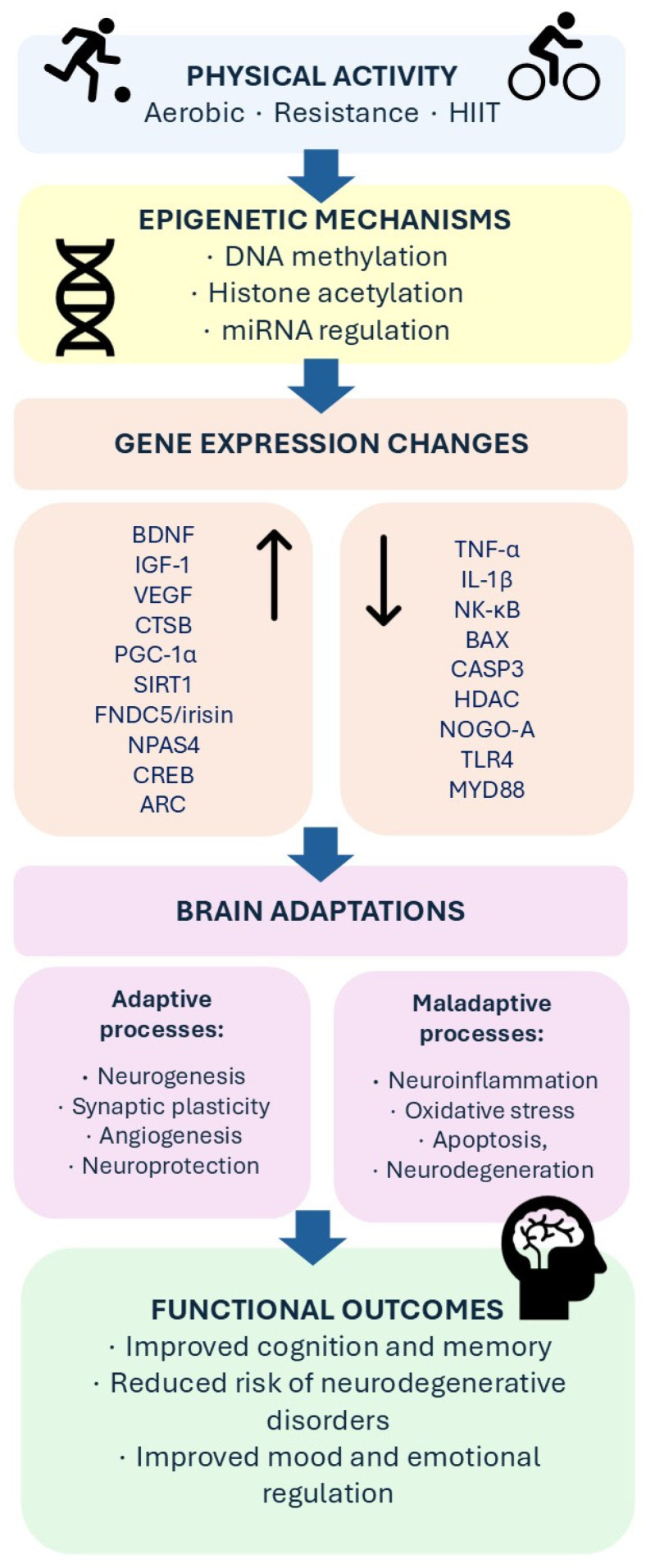
Schematic overview of how physical activity influences brain plasticity and cognitive function through epigenetic regulation, coordinated gene expression changes, and adaptive neurobiological responses. The figure is intended as a conceptual framework based on the available evidence and should not be interpreted as implying direct or uniformly established causal relationships for all depicted pathways. Upward and downward arrows denote upregulation and downregulation of gene expression, respectively.

Epigenetics may also integrate metabolic and neuronal pathways activated during exercise. Evidence shows that exercise-induced metabolic factors, including nicotinamide adenine dinucleotide (NAD+)-dependent signalling and sirtuin activity, can influence chromatin remodeling and transcriptional regulation in neurons. For instance, animal data indicate that exercise may enhance hippocampal plasticity through SIRT1-dependent upregulation of the PGC-1α/FNDC5/BDNF axis [44,45,46]. These mechanisms provide a molecular link between systemic metabolic adaptations to physical activity and long-term changes in gene expression associated with neuronal plasticity and cognitive function. These findings suggest that epigenetics is not an isolated layer of exercise adaptation but rather a key regulatory interface linking physical activity to broader neurogenomic signalling networks. Through epigenetic changes, exercise may shape both the activation of genes that support brain plasticity and the suppression of pathways that constrain neuronal adaptation. A schematic overview of the proposed mechanisms linking physical activity, epigenetic regulation, changes in gene expression, brain adaptations, and functional outcomes is presented in Figure 1.

## 5. Neurogenomic Signalling Pathways

Exercise-induced brain plasticity arises from an integrated neurogenomic response that translates neuronal activity and systemic metabolic signals generated during physical activity into sustained transcriptional adaptations in the brain. In this context, epigenetics serves as a mechanistic interface linking exercise-related stimuli to long-term remodeling of gene expression in the brain. These molecular pathways can be divided into upregulated mediators that support neuroplasticity, metabolic adaptation, muscle–brain communication, and angiogenesis, and downregulated pathways linked to neuroinflammation, oxidative stress, and apoptotic signalling [3]. Crucial genes activated by exercise are summarized in Table 1, whereas selected genes and molecular pathways suppressed by exercise are presented in Table 2. From this perspective, neuroplasticity should not be viewed only as the consequence of increased neurotrophic signalling, but rather as the result of an integrated regulatory shift that affects both adaptive and maladaptive programs. It should be noted that much of the mechanistic evidence discussed in this section derives from animal models, whereas direct human evidence remains comparatively limited. Moreover, the level of evidence is not uniform across pathways, as some mechanisms are supported by both animal and human studies, whereas others remain based primarily on experimental data from rodent models. Likewise, these mechanisms are not necessarily shared to the same extent across exercise modalities, because some findings derive from aerobic or voluntary exercise paradigms, whereas evidence from resistance, HIIT, or forced exercise models remains more limited or less consistent for several pathways.

Activity-dependent transcription represents a fundamental mechanism linking neuronal activity to long-term changes in gene expression. Synaptic activation triggers intracellular signalling cascades that convert electrical activity into transcriptional responses, regulating synaptic plasticity and memory formation. One of the most important transcription factors involved in this process is CREB. Synaptic stimulation leads to calcium influx through N-methyl-D-aspartate (NMDA) receptors and voltage-gated calcium channels, activating pathways such as calcium/calmodulin-dependent protein kinase type IV (CaMKIV), mitogen-activated protein kinase/extracellular signal-regulated kinase (MAPK/ERK), and protein kinase A (PKA). These kinases phosphorylate CREB at Ser133, enabling CREB to recruit transcriptional co-activators such as CREB-binding protein (CBP) and initiate transcription of genes involved in synaptic plasticity and neuronal survival [59]. Genetic studies in mice demonstrated that disruption of CREB-dependent transcription severely impairs long-term memory formation, highlighting the central role of this pathway in neuronal plasticity. One of the most prominent targets of CREB signalling is BDNF, a neurotrophin that plays a key role in neuronal survival, dendritic growth, and synaptic strengthening. BDNF expression is strongly regulated by neuronal activity and CREB-dependent transcription in the hippocampus. Following release, BDNF binds to its high-affinity receptor, tropomyosin receptor kinase B (TrkB), activating intracellular signalling cascades, including the phosphoinositide 3-kinase (PI3K)-protein kinase B (Akt), MAPK-ERK, and phospholipase C-gamma (PLCγ), that promote synaptic plasticity and long-term potentiation. Experimental animal studies have demonstrated that physical exercise significantly increases BDNF expression in the hippocampus and enhances learning and memory performance, suggesting that activity-dependent transcriptional pathways represent a major molecular mechanism underlying exercise-induced brain plasticity [49,54]. Activity-dependent regulation of plasticity-related genes may also involve MeCP2, which links DNA methylation to transcriptional control and has been implicated in synaptic plasticity and neuronal gene regulation, including activity-dependent modulation of BDNF expression [30].

Physical exercise also activates metabolic regulators that influence neuronal gene expression. The main regulator involved in these processes is PGC-1α, a transcriptional coactivator that coordinates mitochondrial biogenesis, oxidative metabolism, and cellular energy homeostasis. Rather than acting simply as a classical neuroplasticity-related mediator, PGC-1α may be better understood as a metabolic integrator that links peripheral exercise responses to central neurotrophic and transcriptional adaptations. This is particularly evident in the PGC-1α–FNDC5/irisin axis, through which skeletal muscle activity may influence hippocampal BDNF expression [11,56]. Wrann and colleagues demonstrated that exercise increases skeletal muscle expression of PGC-1α, which induces transcription of the membrane FNDC5. Processing of this protein generates the circulating myokine irisin, which crosses the blood–brain barrier and stimulates *BDNF* expression in the hippocampus. This pathway represents a key mechanism linking peripheral metabolic signals generated during exercise to central neurotrophic responses in the brain, illustrating that exercise-induced brain plasticity cannot be understood exclusively as a local neuronal phenomenon, but also as a consequence of systemic metabolic communication [11].

Angiogenic pathways are also activated in response to exercise, promoting vascular remodeling and increased cerebral blood flow. Among the central mediators of this response is VEGF, which is upregulated by hypoxia-inducible factor-1α (HIF-1α) and metabolic stress during exercise. VEGF signalling through its receptor, VEGFR-2, activates downstream pathways, including PI3K-Akt and MAPK, promoting endothelial cell proliferation, angiogenesis, and vascular remodelling. In the hippocampus, angiogenesis is closely coupled with neurogenesis, suggesting that exercise-induced vascular adaptations contribute to the molecular environment that supports neuronal plasticity and cognitive function [10,54]. This relationship has been shown most clearly in animal models.

In addition to activating neuroplasticity-related genes, physical exercise induces downregulation of gene expression, particularly in pathways associated with neuroinflammation, oxidative stress, and apoptosis. Strong evidence indicates that regular physical activity suppresses neuroinflammatory signalling pathways in the CNS, leading to reduced expression or activity of key pro-inflammatory mediators, including tumor necrosis factor alpha (TNF-α), interleukin 1 beta (IL1B), interleukin 6 (IL6), and the transcription factor nuclear factor kappa B (NFKB1/NF-κB), all of which have been implicated in inflammation-related neuronal dysfunction. These anti-inflammatory effects are particularly relevant in the hippocampus, where chronic neuroinflammation has been linked to impaired neurogenesis and cognitive decline. In aged animal models, exercise shifted the hippocampal inflammatory profile toward a less pro-inflammatory state, as reflected by lower TNF-α/IL-10, IL1B/IL-10, and IL6/IL-10 ratios. Other studies have shown that physical activity reduces basal microglial activation and attenuates inflammatory signalling in the aging brain. Together, these findings suggest that exercise promotes a more permissive molecular environment for neuronal survival and plasticity, not only by inducing neurotrophic pathways, but also by suppressing gene networks that sustain neuroinflammation [1,60,61]. In parallel, exercise has been shown to attenuate apoptotic signalling pathways, as reflected by reduced expression of pro-apoptotic genes such as *BAX* and *CASP3*, thereby promoting neuronal survival and resilience [62,63]. This shift toward an anti-apoptotic molecular profile helps preserve neuronal integrity, particularly under conditions of aging or neurodegenerative stress. These transcriptional adaptations also involve suppression of molecular pathways that functionally constrain synaptic plasticity. For example, decreased HDAC activity has been associated with enhanced chromatin accessibility and increased expression of plasticity-related genes [35,64]. Similarly, exercise-related attenuation of axonal growth inhibitors such as NOGO-A may facilitate structural remodeling and synaptic reorganization within neural circuits [65]. Selected examples of genes and molecular pathways suppressed by exercise are summarized in Table 2. These molecular adaptations are particularly relevant in the context of brain aging, neurodegenerative disorders, and mental health, where impaired plasticity, chronic inflammation, and metabolic dysregulation contribute to disease vulnerability.

**Table 2:** Selected genes and molecular pathways downregulated by exercise and involved in brain plasticity.

**Gene Symbol** **(** **Full Gene Name** **)**	**Chromosomal Location**	**Molecular Role in the Brain**	**Exercise**-**Induced Effect**	**Functional Outcome**	**Main Brain Region Affected**	**Type of Evidence**	**Key References**
*BAX* (BCL2 Associated X Protein)	19q13.33	Pro-apoptotic regulator of neuronal cell death	Exercise suppresses BAX-related apoptotic signalling and reduces Bax expression in experimental models of brain injury and neurodegenerative stress	Increased neuronal survival and reduced hippocampal apoptosis	Hippocampus	Animal	[66,67,68]
*CASP3* (Caspase 3)	4q35.1	Central executor of apoptosis involved in neuronal cell death under pathological stress	Exercise suppresses CASP3-related apoptotic signalling and reduces caspase-3 expression/activation in experimental models of hippocampal injury and neurodegenerative stress	Reduced neuronal apoptosis and improved neuronal survival	Hippocampus	Animal	[66,67,69]
*HDAC* (Histone Deacetylase)	–	Epigenetic repressor of synaptic plasticity genes	Exercise decreases hippocampal histone deacetylase activity and shifts the HAT/HDAC balance toward a more transcriptionally permissive state	Increased chromatin accessibility and facilitation of plasticity-related gene expression	Hippocampus	Animal	[35,70]
*IL1B* (Interleukin 1 Beta)	2q14.1	Mediator of neuroinflammatory responses and cognitive impairment	Exercise attenuates IL1B-associated inflammatory signalling in the brain	Reduced inflammation and a more permissive environment for neurogenesis	Hippocampus	Animal	[60,71]
*MYD88* (Myeloid Differentiation Primary Response 88)	3p22.2	Adaptor protein mediating TLR4-triggered inflammatory cascades	Exercise attenuates MyD88-dependent inflammatory signalling in experimental models of hippocampal dysfunction and brain injury	Reduced downstream inflammatory activation and improved neuronal milieu	Hippocampus, brain tissue	Animal	[47,72,73]
*NFKB1* (Nuclear Factor Kappa B Subunit 1)	4q24	Transcription factor controlling inflammatory gene expression	Exercise suppresses NF-κB-related neuroinflammatory signalling pathways	Reduced inflammatory transcriptional activity and support for hippocampal neurogenesis	Hippocampus	Animal	[53,72]
*RTN4*/*NOGO-A* (Reticulon 4)	2p16.3	Myelin-associated inhibitor of axonal growth and synaptic remodeling	Exercise attenuates NOGO-A-related inhibitory signalling and may transiently reduce Nogo-A expression in experimental models of brain injury and motor learning	Reduced molecular constraints on axonal remodeling and enhanced plasticity	Cortex, peri-infarct cortex	Animal	[74,75]
*TLR4* (Toll Like Receptor 4)	9q33.1	Upstream innate immune receptor activating MyD88/NF-κB inflammatory signalling in the brain	Exercise downregulates TLR4-associated neuroinflammatory signalling in experimental models of hippocampal dysfunction and brain injury	Reduced neuroinflammation and improved neuronal milieu conducive to hippocampal plasticity	Hippocampus, brain tissue	Animal	[53,72,73]
*TNF* (Tumor Necrosis Factor)	6p21.33	Pro-inflammatory cytokine regulating neuroinflammation and synaptic dysfunction	Exercise reduces TNF-related inflammatory signalling and shifts the hippocampal cytokine balance toward a less pro-inflammatory state	Reduced neuroinflammation and improved synaptic function	Hippocampus	Animal	[1,60]

In addition to exercise-responsive signalling pathways, gene–environment interactions may also contribute to inter-individual variability in neuroplastic and cognitive responses to physical activity. In this context, the available evidence comes primarily from human association and intervention studies, although the mechanistic basis of these relationships remains less well established than in experimental animal models. In particular, common polymorphisms in genes related to neurotrophic signalling and neurotransmission systems, such as *BDNF* and selected dopaminergic and serotonergic pathway-related genes, may partly influence phenotypic adaptations, including cognition, functional responses, and responsiveness to training. For example, the *BDNF* Val66Met (rs6265) polymorphism has been associated with variability in exercise-related changes in peripheral BDNF levels and in cognitive function. In older adults, Val/Val homozygotes (GG) may derive greater cognitive benefit from physical activity, whereas in elderly individuals with mild cognitive impairment, Met carriers (GA/AA) have shown significant exercise-related increases in peripheral protein levels [76,77,78]. In the dopaminergic system, human genetic studies have implicated *ANKK1*/*DRD2* rs1800497 (TaqIA) and *DRD2* rs6277 in individual differences in exercise-related reinforcement and responsiveness to physical activity interventions, with some evidence suggesting a role of the G allele of rs1800497 [79,80]. Likewise, human studies have linked variants in genes of the serotonergic pathway to sport-related phenotypes, suggesting that genetic background may partly contribute to inter-individual differences in exercise responsiveness [80,81]. This perspective is especially relevant in the translational context, as it suggests that the neurobiological effects of exercise may depend not only on the training stimulus itself, but also on the individual genetic background.

## 6. Exercise, Brain Aging, Neurodegeneration, and Mental Health

Brain aging is accompanied by progressive declines in hippocampal volume, synaptic plasticity, cerebral perfusion, and cognitive performance, particularly in domains such as memory and executive function. Physical activity has emerged as a promising non-pharmacological strategy that may attenuate several of these age-related changes. Evidence in this area comes from both animal models and human observational or interventional studies, although the mechanistic depth is generally greater in animal research. In older adults, higher aerobic fitness has been associated with larger hippocampal volumes and better spatial memory, and a randomized controlled trial further showed that one year of aerobic exercise increased hippocampal volume and improved memory performance, suggesting that exercise may partly counteract age-related structural brain loss [4,82]. Experimental studies also show that exercise can restore age-sensitive aspects of hippocampal plasticity. In aged mice, voluntary running increased hippocampal neurogenesis and improved learning and memory, demonstrating that the aging brain retains substantial capacity for structural adaptation when exposed to physical activity. Similar findings were reported in middle-aged mice, in which exercise enhanced the proliferation of neural stem cells, neurite growth, and survival of neuronal progenitor cells in the dentate gyrus [83,84]. This is especially relevant because age-related decline in brain function appears to reflect not only structural changes, but also progressive dysregulation of molecular pathways that normally support neuronal plasticity and resilience.

These observations are especially relevant in the context of neurodegenerative disorders, where aging-related reductions in plasticity interact with disease-specific molecular pathology. In this context, the epigenetic and neurogenomic mechanisms discussed above, particularly those involving BDNF-related signalling, neuroinflammatory regulation, mitochondrial function, and activity-dependent transcription, may help explain how exercise supports neuronal resilience in Alzheimer’s disease. Exercise has been linked to beneficial effects on cognition and brain structure. A 26-week randomized controlled trial in individuals with early Alzheimer’s disease found that aerobic exercise was feasible and improved functional ability. In contrast, subsequent analyses from exercise trials suggested that physical activity may reduce the rate of cognitive decline in mild-to-moderate disease [85,86]. Large prospective cohort studies and meta-analyses further support the protective role of physical activity against cognitive decline and dementia. Epidemiological research suggests that individuals who engage in regular physical activity have a significantly lower risk of developing Alzheimer’s disease and other forms of dementia compared with physically inactive individuals [85,86,87]. More recent longitudinal studies have also demonstrated that higher levels of physical activity are associated with reduced incidence of dementia and slower cognitive decline in aging populations [88,89]. In mouse models, physical activity has been shown to preserve hippocampal neurogenesis even in the presence of amyloid pathology, although the magnitude of this effect is influenced by age. Other experimental studies reported that running exercise prevented amyloid-β-induced cognitive impairment, supporting the view that exercise may modulate both neuronal resilience and pathological processes relevant to neurodegeneration [90,91]. Exercise has also shown promise in Parkinson’s disease, where neurodegeneration affects dopaminergic pathways and motor–cognitive integration. This is consistent with the pathways outlined in Section 4 and Section 5, as exercise may influence dopaminergic circuit function through neurotrophic support, synaptic plasticity, mitochondrial regulation, and the attenuation of neuroinflammatory and apoptotic signalling. Human studies suggest that structured exercise may improve both motor and cognitive features of Parkinson’s disease, while imaging data from treadmill training have shown increased striatal dopamine D2 receptor binding potential in early Parkinson’s disease, consistent with exercise-induced neuroplastic adaptations in dopaminergic signalling [92,93]. Experimental studies further propose that exercise may exert neuroprotective effects on dopaminergic neurons by enhancing neurotrophic signalling and synaptic plasticity within basal ganglia circuits [65]. Exercise has also been shown to influence molecular processes involved in Parkinson’s disease pathology, including mitochondrial function, and α-synuclein-related neurodegenerative mechanisms [94].

Physical activity is also increasingly recognized as a useful strategy in mood disorders. These effects may be linked to exercise-induced regulation of neurotrophic pathways, epigenetic modulation of plasticity-related genes, and the suppression of inflammatory processes that negatively affect neuronal function and emotional regulation. Depression has been associated with structural and functional alterations in brain regions involved in emotional functioning and cognition, including reduced hippocampal volume, impaired neurogenesis, and dysregulation of neurotrophic pathways. Several clinical studies and meta-analyses suggest that regular physical activity may reduce depressive symptoms and improve cognitive and emotional functioning in individuals with depression [4,95]. These antidepressant effects may be mediated by mechanisms similar to those observed in exercise-induced brain plasticity, including increased hippocampal neurogenesis, enhanced BDNF signalling, and improved synaptic plasticity [96]. These findings support the concept that physical activity may contribute to resilience not only against neurodegenerative processes but also against stress-related neuropsychiatric disorders. In addition to effects on neuronal plasticity and mood regulation, physical activity may also modulate neuroinflammatory processes associated with brain aging. Aging is characterized by increased microglial activation and elevated pro-inflammatory cytokine levels in the brain, which can contribute to synaptic dysfunction and neuronal damage. Experimental research suggests that regular exercise can attenuate these inflammatory responses and promote a more neuroprotective microglial phenotype, thereby supporting neuronal health during aging [60,61]. Another mechanism by which exercise may influence brain aging involves regulating mitochondrial function and cellular metabolism. Age-related mitochondrial dysfunction and oxidative stress are considered major contributors to neurodegeneration. Physical activity has been shown to enhance mitochondrial biogenesis and improve cellular energy metabolism [62,63]. These observations support the relevance of exercise-responsive molecular pathways across aging and disease contexts, while also underscoring remaining questions about their temporal dynamics, cell-type specificity, and translational relevance in humans [4,85,92].

## 7. Future Directions in Neurogenomics of Exercise

Future progress in neurogenomics will depend on moving beyond single-pathway models toward integrated multi-omics approaches that capture the complexity of exercise-induced adaptations across tissues and over time. Large-scale initiatives, such as the Molecular Transducers of Physical Activity Consortium (MoTrPAC), were designed precisely to generate such molecular maps, combining transcriptomic, proteomic, metabolomic, and other omics layers in both animal and human studies. Although not focused exclusively on the brain, these frameworks are highly relevant to neurogenomics because they provide a model for linking systemic exercise signals with tissue-specific molecular responses [97,98]. Within this broader context, transcriptomics is likely to remain a central tool for defining exercise-responsive molecular programs in the brain. RNA sequencing (RNA-seq) has already shown that exercise alters hippocampal gene-expression profiles related to metabolism, synaptic plasticity, and neurogenesis. In contrast, combined RNA-seq and whole-genome bisulfite sequencing has demonstrated that endurance and resistance exercise can reshape both the hippocampal transcriptome and epigenome in mice. More recently, single-cell transcriptomic analysis has provided cell-type-resolved evidence that exercise affects hippocampal cellular composition and may accelerate maturation of specific neuronal subpopulations. These findings suggest that future work should increasingly focus on cell-specific and circuit-specific transcriptional responses rather than treating the brain as a homogeneous tissue [7,8].

At the same time, a significant limitation of the current literature is that most mechanistic studies in exercise neurogenomics have been conducted in animal models, particularly rodents. In contrast, direct evidence from humans remains comparatively limited and often relies on indirect approaches, such as neuroimaging and peripheral molecular markers [5]. This distinction is important because differences in brain structure, lifespan, exercise paradigms, and the feasibility of direct tissue sampling may limit the direct translation of mechanistic findings from animal models to humans. In addition, many human studies are constrained by small sample sizes, heterogeneous exercise protocols, and the reliance on peripheral biomarkers that may not fully reflect molecular processes occurring in the brain. Although these models have provided essential insights into hippocampal plasticity, neuroinflammatory signalling, and epigenetics, their direct translation to humans remains limited. Future research should therefore place greater emphasis on well-controlled human studies integrating molecular profiling, neuroimaging, physiological phenotyping, and cognitive assessment to determine whether the neurogenomic mechanisms identified in animal models are conserved and functionally relevant in the human brain.

Another major direction concerns the integration of epigenetic profiling with transcriptomic and physiological data. Exercise-induced changes in DNA methylation, chromatin accessibility, histone modifications, and ncRNAs are unlikely to act in isolation; rather, they form interconnected regulatory layers that shape long-term transcriptional adaptation. Future studies should therefore combine epigenomic and transcriptomic datasets collected from the same samples and time points, ideally across different exercise modalities, ages, and sexes. This is especially important in the brain, where the temporal dynamics of plasticity-related gene regulation may differ markedly between acute and chronic exercise exposure [7,8].

An important unresolved issue also concerns the dose–response relationship between exercise and neurogenomic or epigenetic adaptations in the brain. Available evidence suggests that molecular responses may vary according to exercise modality, intensity, duration, frequency, and cumulative training volume, and that acute and chronic exercise exposures may induce partly distinct effects. However, the current literature remains too heterogeneous to define a precise exercise dose required to elicit measurable epigenetic or transcriptional changes in the brain, particularly in humans. Addressing this question will require more standardized study designs that integrate molecular profiling with carefully characterized exercise protocols.

Another underexplored area concerns epigenetic supplements and nutrition-based modulators that may interact with exercise-responsive neurogenomic pathways. At present, direct research in exercise neurogenomics is limited, but several adjacent lines of research suggest this is a promising direction. Brain-focused studies have shown that dietary polyphenols can modify hippocampal expression of epigenetic regulators, including DNMTs and TET enzymes. At the same time, work on methyl donors indicates that nutrients involved in one-carbon metabolism can influence brain-relevant epigenetics. In parallel, the DO-HEALTH trial reported that omega-3 supplementation, with additive effects when combined with vitamin D and exercise, modestly slowed DNA methylation clock measures of biological aging in older adults. Although these findings are not specific to exercise-induced brain plasticity, they support the idea that future studies should examine whether diet-derived epigenetic modifiers can enhance or limit the neurogenomic response to exercise [99,100,101].

A further priority is the development of personalized exercise medicine. Inter-individual variability in exercise response is well established, and future neurogenomic studies should move toward identifying the molecular signatures that predict who benefits most from specific exercise modalities, intensities, and durations. This will likely require combining genetic background, epigenetic status, baseline fitness, age, sex, circadian factors, and lifestyle exposures into individualized response models. In addition, genetic variation may partly modulate individual responsiveness to exercise-induced neuroplastic and cognitive adaptations, further supporting the rationale for precision exercise medicine. Conceptual and methodological frameworks for personalized exercise prescription have already been proposed, and recent work continues to emphasize that understanding variability in biological responses is essential for precision exercise medicine. In the neurogenomic context, this approach may ultimately allow exercise interventions to be tailored for cognitive aging, neurodegenerative risk, or recovery after neurological injury [102,103,104]. This perspective may also have practical implications for neurological rehabilitation and the optimization of athletic performance. In neurological rehabilitation, a better understanding of epigenetic and neurogenomic mechanisms may inform the design of more individualized exercise interventions to enhance cognitive recovery, neural plasticity, and functional outcomes after neurological injury or disease. In athletes, integrating such data into exercise models may help refine training strategies to enhance cognitive resilience, motor learning, stress adaptation, and broader neurobiological responses to training. Although these applications remain largely prospective, they align closely with the goals of precision medicine and represent an important direction for future translational research [102,103,104,105,106].

Two additional priorities also deserve emphasis. First, future studies should invest in longitudinal, standardized phenotyping, including repeated molecular sampling, neuroimaging, and cognitive assessment, so that mechanistic findings can be tied to meaningful functional outcomes. Second, more work is needed on causal validation, not just correlation. High-throughput discovery must be followed by mechanistic testing in experimental systems to determine which transcripts, epigenetic marks, and signalling pathways are truly required for exercise-induced neuroplasticity. In practical terms, the field is now moving from descriptive omics toward integrative and ultimately predictive neurobiology [8,97,98].

The future of exercise neurogenomics lies in integrating multi-omics, cell-type-resolved transcriptomics, epigenetic profiling, nutrition-sensitive modulators, and precision exercise frameworks into unified experimental designs. Machine learning approaches may help integrate datasets to identify predictive molecular signatures of exercise responsiveness. Such an approach offers the best chance of explaining how physical activity produces heterogeneous but biologically meaningful effects on brain plasticity, cognitive function, and resilience to aging and neurodegeneration [7,102]. Addressing these challenges will be essential for moving the field from descriptive models of exercise responsiveness toward a more predictive and mechanistically grounded understanding of brain adaptation.

## 8. Conclusions

Current evidence suggests that physical activity influences brain plasticity through interacting epigenetic, transcriptional, metabolic, and inter-organ signalling mechanisms. In this context, exercise-induced neuroplasticity seems to depend not only on the activation of adaptive pathways that promote neurogenesis, synaptic plasticity, angiogenesis, and metabolic adaptation, but also on the suppression of maladaptive processes associated with neuroinflammation, oxidative stress, and apoptosis. The balance between these responses may be as important as the magnitude of change in any single molecular mediator. Progress in this field will depend on combining mechanistic, translational, and personalized approaches to better understand how exercise-responsive neurogenomic pathways support brain health throughout life. In the longer term, such knowledge may contribute to the development of personalized exercise medicine for cognitive health and the prevention of neurodegenerative diseases.

## Figures and Tables

**Table 1:** Selected genes and molecular pathways upregulated by exercise and involved in brain plasticity.

Gene Symbol (Full Gene Name)	Chromosomal Location	Molecular Role in the Brain	Exercise-Induced Effect	Functional Outcome	Main Brain Region Affected	Type of Evidence	Key References
*ARC* (Activity-Regulated Cytoskeleton Associated Protein)	8q24.3	Immediate early gene involved in synaptic plasticity and memory consolidation	Exercise increases neuronal activity and *ARC* expression	Regulation of synaptic strength and long-term memory formation	Hippocampus, cortex	Animal	[47,48]
*BDNF* (Brain-Derived Neurotrophic Factor)	11p14.1	Major neurotrophin regulating neuronal survival, neurogenesis, and synaptic plasticity	Exercise increases *BDNF* expression in the hippocampus and circulation	Improved learning, memory, and neuroplasticity	Hippocampus, cortex	Animal, human	[2,4,49]
*CREB1* (cAMP Response Element-Binding Protein 1)	2q34	Transcription factor controlling genes involved in neuronal plasticity and memory formation	Exercise activates CREB signalling pathways in neurons	Enhanced transcription of plasticity-related genes	Hippocampus	Animal, limited human evidence	[50,51]
*FNDC5* (Fibronectin Type III Domain Containing 5)	1p35.1	Precursor of the myokine irisin mediating muscle–brain communication	Exercise stimulates *FNDC5* expression and irisin release from skeletal muscle	Induction of BDNF expression and neuroplasticity	Hippocampus	Animal, human	[11,52,53]
*IGF1* (Insulin-Like Growth Factor 1)	12q23.2	Growth factor supporting neuronal survival and neurogenesis	Exercise increases circulating IGF-1, which can cross the blood–brain barrier	Enhanced neurogenesis and neuronal resilience	Hippocampus	Animal, human	[9,54]
*NPAS4* (Neuronal PAS Domain Protein 4)	11p15.4	Activity-dependent transcription factor regulating inhibitory synapse development	Exercise-related neuronal activity may stimulate *NPAS4* expression	Homeostatic regulation of excitatory–inhibitory balance	Hippocampus	Animal	[55]
*PPARGC1A* (Peroxisome Proliferator-Activated Receptor Gamma Coactivator 1-Alpha)	4p15.2	Transcriptional coactivator regulating mitochondrial biogenesis and metabolic signalling	Exercise induces PGC-1α protein expression in skeletal muscle and, in animal models, may also enhance hippocampal metabolic signalling	Activation of the FNDC5/irisin pathway and support of metabolic adaptations linked to brain plasticity	Indirect brain effects (including hippocampal signalling)	Animal, indirect human evidence	[11,56]
*SIRT1* (Sirtuin 1)	10q21.3	NAD+-dependent deacetylase regulating metabolism, stress resistance, and neuronal survival	Exercise activates SIRT1 signalling linked with mitochondrial adaptation	Neuroprotection and improved neuronal metabolism	Hippocampus	Animal	[44,57]
*VEGFA* (Vascular Endothelial Growth Factor A)	6p21.1	Angiogenic factor regulating vascular growth and neurogenesis	Exercise stimulates *VEGFA* expression and cerebral angiogenesis	Improved vascularization and support of hippocampal neurogenesis	Hippocampus	Animal, human	[10,58]

## Data Availability

No new data were created or analyzed in this study. Data sharing does not apply to this article.

## Abbreviations

The following abbreviations are used in this manuscript:

Akt	Protein kinase B
ARC	Activity-regulated cytoskeleton-associated protein
ASC	Apoptosis-associated speck-like protein containing a CARD
BAX	BCL2-associated X protein
BDNF	Brain-derived neurotrophic factor
CaMKIV	Calcium/calmodulin-dependent protein kinase IV
CASP3	Caspase 3
CBP	CREB-binding protein
CNS	Central nervous system
CpG	Cytosine–phosphate–guanine
CREB	cAMP response element-binding protein
CREB1	cAMP response element-binding protein 1
DNA	Deoxyribonucleic acid
DNMTs	DNA methyltransferases
DO-HEALTH	Vitamin D3–Omega-3–Home Exercise–Healthy Ageing and Longevity Trial
ERK	Extracellular signal-regulated kinase
FNDC5	Fibronectin type III domain-containing protein 5
HAT	Histone acetyltransferase
HDAC	Histone deacetylase
HIF-1α	Hypoxia-inducible factor 1-alpha
HIIT	High-intensity interval training
IGF-1	Insulin-like growth factor 1
IL-10	Interleukin 10
IL-1B	Interleukin 1 beta
IL-6	Interleukin 6
MAPK	Mitogen-activated protein kinase
mCH	Non-CG methylation
MeCP2	Methyl-CpG-binding protein
miRNA	MicroRNA
MoTrPAC	Molecular Transducers of Physical Activity Consortium
mRNA	Messenger RNA
MYD88	Myeloid differentiation primary response 88
NAD+	Nicotinamide adenine dinucleotide
ncRNA	Non-coding RNA
NF-κB	Nuclear factor kappa B
NFKB1	Nuclear factor kappa B subunit 1
NMDA	N-methyl-D-aspartate
NOGO-A	Neurite outgrowth inhibitor A
NPAS4	Neuronal PAS domain protein 4
Nr3c1	Nuclear receptor subfamily 3 group C member 1
PGC-1α	Peroxisome proliferator-activated receptor gamma coactivator 1-alpha
PI3K	Phosphoinositide 3-kinase
PKA	Protein kinase A
PLCγ	Phospholipase C-gamma
PRISMA	Preferred Reporting Items for Systematic Reviews and Meta-Analyses
RNA-seq	RNA sequencing
RTN4	Reticulon 4
SIRT1	Sirtuin 1
TET	Ten–eleven translocation enzymes
TGF-β	Transforming growth factor beta
TLR4	Toll-like receptor 4
TNF-α	Tumor necrosis factor alpha
TrkB	Tropomyosin receptor kinase B
VEGF	Vascular endothelial growth factor
VEGFA	Vascular endothelial growth factor A
VEGFR-2	Vascular endothelial growth factor receptor 2
